# Correction: Computational capabilities of a multicellular reservoir computing system

**DOI:** 10.1371/journal.pone.0315300

**Published:** 2024-12-05

**Authors:** Vladimir Nikolić, Moriah Echlin, Boris Aguilar, Ilya Shmulevich

There are errors in the Funding statement. The correct Funding statement is as follows: V.N. was supported in part by a scholarship from the UBC Bioinformatics Graduate Program (www.bioinformatics.ubc.ca) via the NSERC (www.nserc-crsng.gc.ca) CREATE program in High-Dimensional Biology. The funders had no role in study design, data collection and analysis, decision to publish, or preparation of the manuscript.
