# Supplementary material for: Computational capabilities of a multicellular reservoir computing system
Source: PLoS One. 2023 Apr 6;18(4):e0282122. doi: 10.1371/journal.pone.0282122 (PMC10079015; doi:10.1371/journal.pone.0282122)
Supplement: S1 File — (PDF) [file pone.0282122.s001.pdf]

Supplementary Data

Section S1: Random Boolean network construction

The total number of edges in a Random Boolean Network (RBN) within a cell is:

$$e = (N - (1 + E)) \cdot 2$$

where N is the total number of genes and E the number of Extracellular Signalling Molecules (ESMs), which is equal to the number of ESM receptor genes. The $1 + E$ part comes from the fact that the input gene (1) and ESM receptors (E) do not have incoming edges. The rest of the genes should have an average in-degree of 2, hence the factor at the end. From e, a subset of edges are used for connections with the input gene. The number of these input edges is modulated with the L parameter, $0 \leq L \leq 1$. The number of input edges is:

$$e_i = (N - (1 + E)) \cdot L$$

To assign the edges to nodes, first, two sets of edges are generated:

1. All possible edges that start at the input gene.

2. All possible edges that do not start at the input gene.

The generated edges exclude those that end in the input gene or ESM receptors. These two sets are randomly shuffled, and from the first set, the first $e_i = (N - (1 + E)) \cdot L$ are selected and assigned. From the second set, the first $e - e_i = (N - (1 + E)) \cdot 2 - (N - (1 + E)) \cdot L$ are selected and assigned. This way, we randomly and uniformly assign edges with an average in-degree of 2 and the exact number of input edges we wanted.

Supplementary tables

Table S1. Proof of Concept fixed parameters. Cells are arranged in a $12x12x12$ cube. All cells are reached by the input signal and all cells are used for output.

Parameter	Value
Gene input fraction (L)	0.5
Reservoir genes (N_r)	100
Cells (C)	$1728(12 \cdot 12 \cdot 12)$
Strains (S)	10
Input layers (I)	*All*
Output layers (O)	*All*
ESM interaction distance (λ)	15.0
ESMs (E)	5
ESM threshold (θ)	11.5
Delay (d)	0
ESM diffusion coefficient (β)	5.0
ESM basal secretion rate	1.0
ESM active secretion rate	5.0
Cell radius	10.0
Voxel side length	20.0
Warm-up time steps (T_W)	100
Total time steps (T)	1000

Table S2. Sensitivity Analysis fixed parameters. All cells are used for output.

Parameter	Value
Output layers (O)	*All*
Delay (d)	0
ESM diffusion coefficient (β)	5.0
ESM basal secretion rate	1.0
ESM active secretion rate	5.0
Cell radius	10.0
Voxel side length	20.0
Warm-up time steps (T_W)	100
Total time steps (T)	1000
Function	*parity*
Window size (w)	7

Table S3. Layered Community fixed parameters. Cells are arranged in three $24x24$ square layers. Only the first layer cells are reached by the input signal and only the last layer cells are used for output.

Parameter	Value
Gene input fraction (L)	0.5
Reservoir genes (N_r)	100
Cells (C)	$1728(24 \cdot 24 \cdot 3)$
Strains (S)	10
Input layers (I)	1
Output Layers (O)	1
ESM interaction distance (λ)	15.0
ESMs (E)	5
ESM threshold (θ)	11.5
Delay (d)	2
ESM diffusion coefficient (β)	5.0
ESM basal secretion rate	1.0
ESM active secretion rate	5.0
Cell radius	10.0
Voxel side length	20.0
Warm-up time steps (T_W)	100
Total time steps (T)	1000
Function	*parity*
Window size (w)	3

Supplementary figures

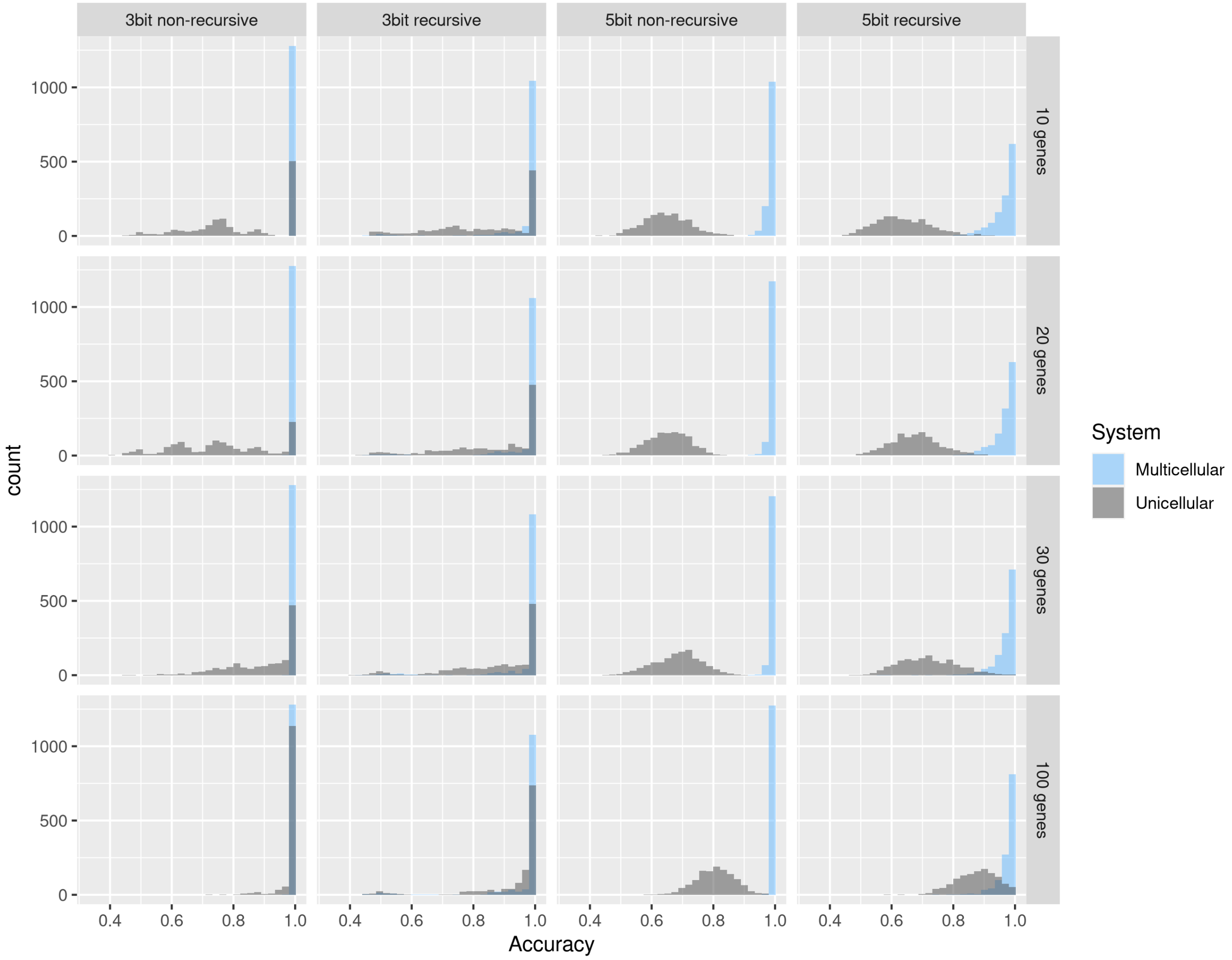

Fig. S1. Comparison between a unicellular system and a multicellular system similar to the Proof of Concept analysis. Here, we test all possible Boolean functions (256) for a 3 bit window, and 256 randomly sampled Boolean functions for a 5 bit window. Additionally, a recursive version of functions is also tested where the least recent input window bit is replaced with the output of the function from the previous timestep. All of the parameters are the same as in the Proof of Concept scenario except for the number of reservoir genes (N_r) per cell which is varied between 10, 20, 30, and 100. Each function was simulated 5 times. In all cases, the multicellular system performs better on average. It is worth noting that in some cases recursive functions perform better than non-recursive for the unicellular system, which is different from the results found in previous work by Echlin et al (1). This is due to different simulation approach between this and the previous work. In this work, simulations last 1,000 timesteps whereas in previous work simulations were only $10 + w - 1$ timesteps long (although many simulations were ran), which we have found to have an effect on the results.

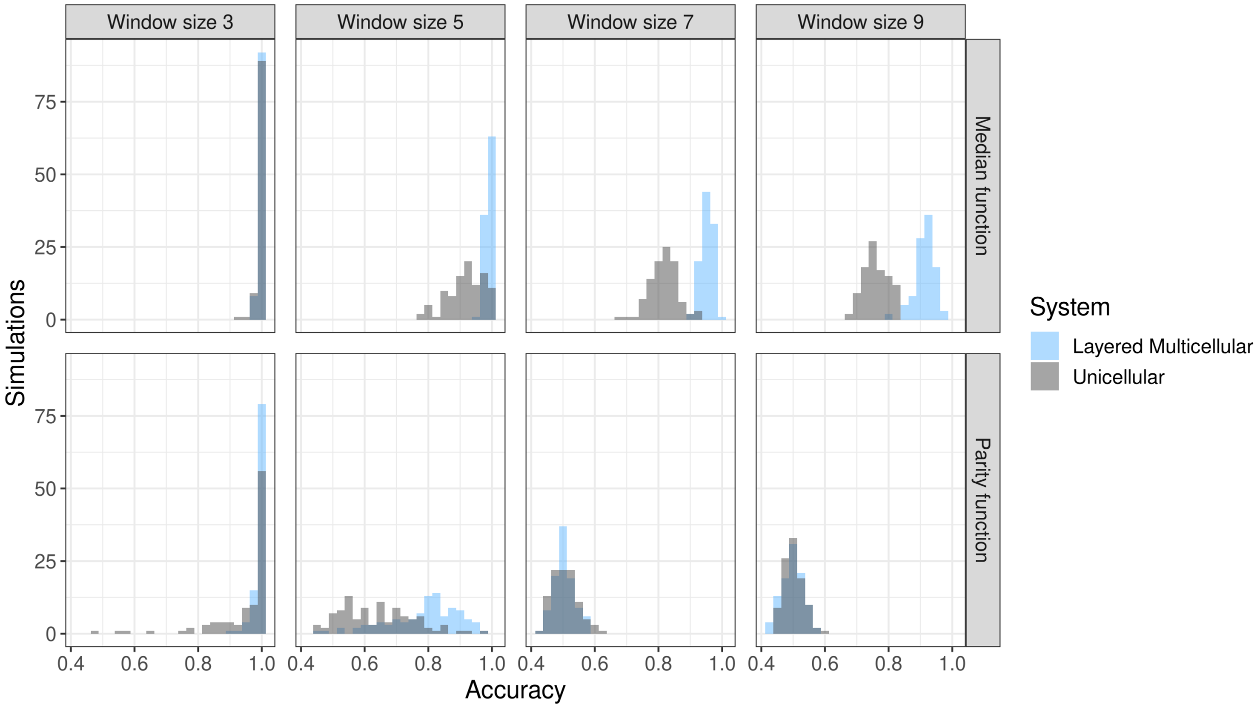

Fig. S2. Comparison between a unicellular system and a Layered Community. In order to maximize accuracy and show the performance capabilities of the Layered community, the number of cells is 7500 and the number of ESMs 20. Each histogram is the accuracy distribution of 100 simulations. Layered Communities outperform unicellular systems, except when both perform equally poorly.

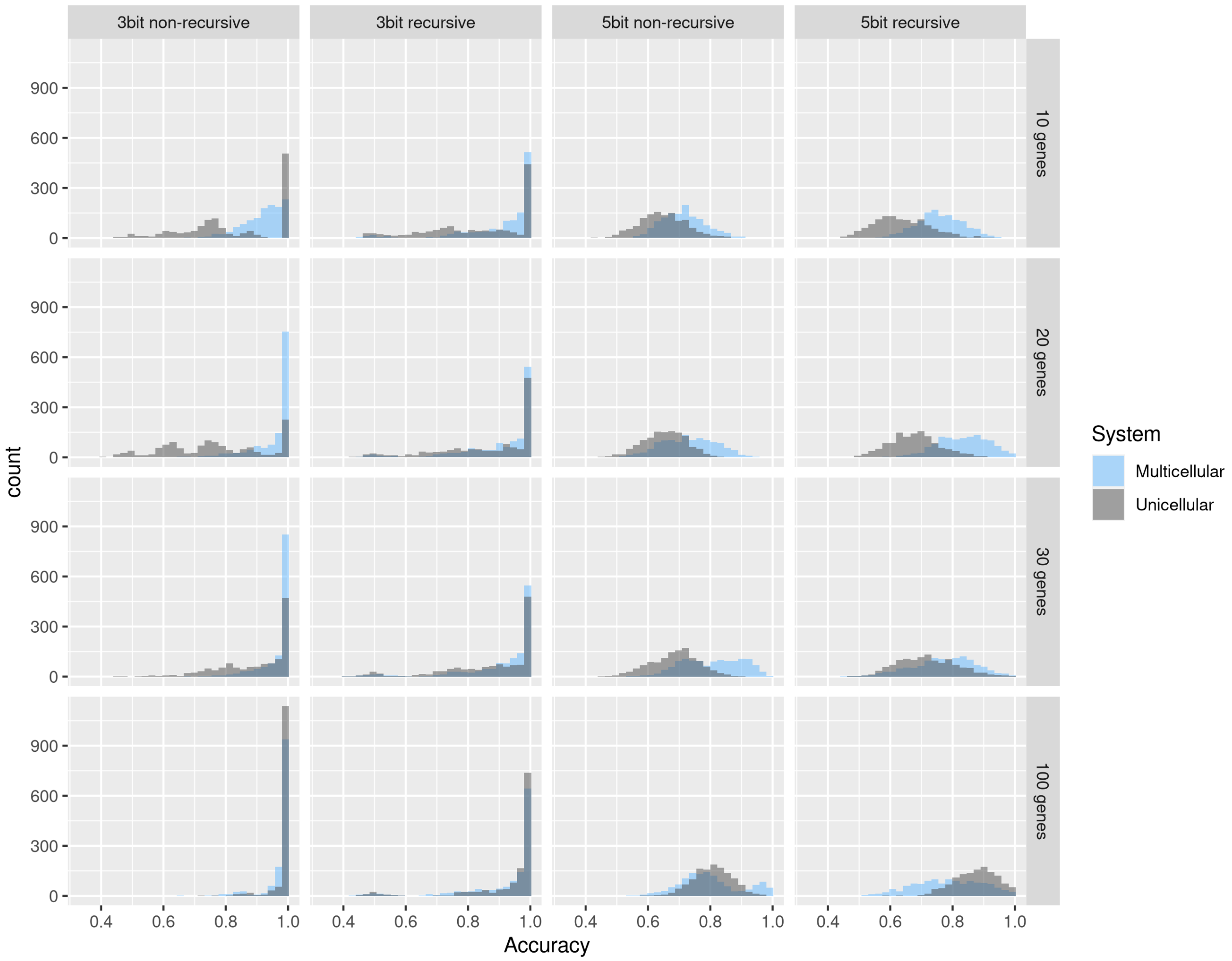

Fig. S3. Comparison between a unicellular system and a multicellular system similar to the Layered Community analysis. Here, we test all possible Boolean functions (256) for a 3 bit window, and 256 randomly sampled Boolean functions for a 5 bit window. Additionally, a recursive version of functions is also tested where the least recent input window bit is replaced with the output of the function from the previous timestep. All of the parameters are the same as in the Layered Community scenario except for the number of reservoir genes (N_r) per cell which is varied between 10, 20, 30, and 100. Each function was simulated 5 times. For lower number of genes, 10, 20, and 30, multicellular system outperforms the unicellular except in case of 3 bit non-recursive functions with 10 genes. At 100 genes, the unicellular and multicellular systems are comparable with unicellular systems at a slight advantage on 5 bit recursive functions.

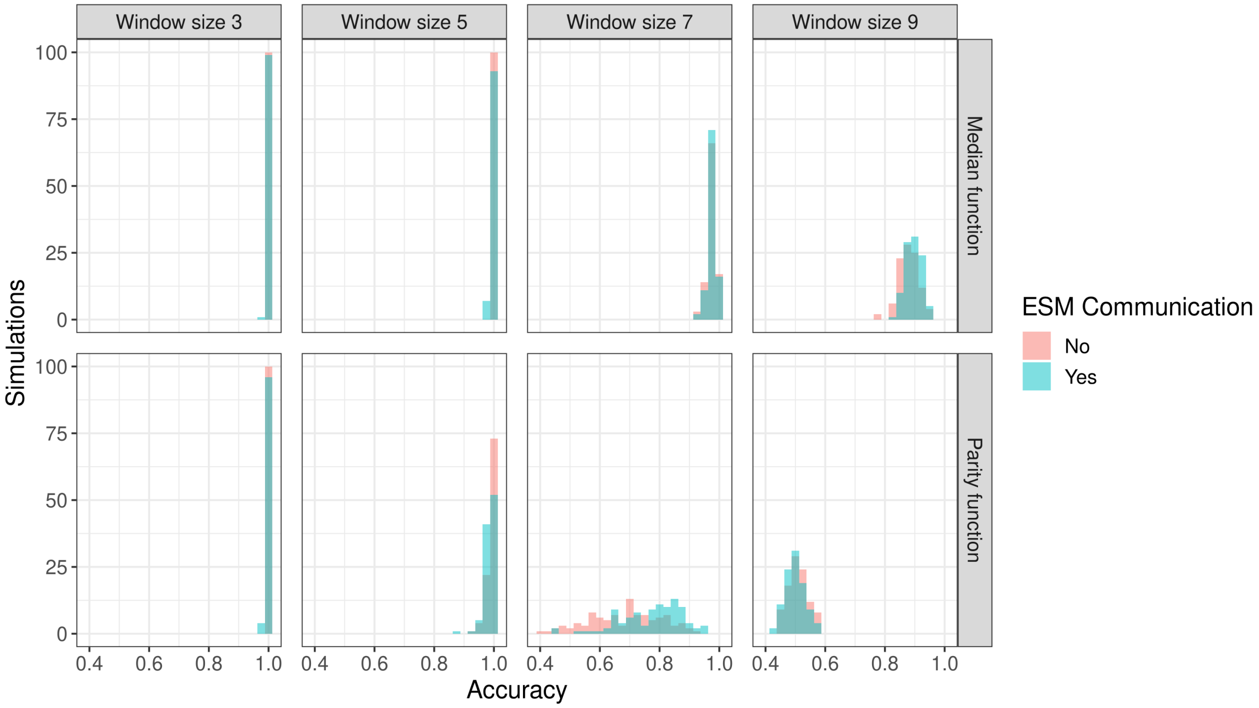

Fig. S4. Comparison between two multicellular systems in which ESM communication is either allowed (bluegreen) or blocked (pink). Simulation parameters are the same as the Proof of Concept simulations (listed in Table S1) with the exception of ESM threshold θ. Disabling ESM communication is achieved by setting the ESM threshold θ to 100000.0. Each histogram is the accuracy distribution of 100 simulations. As the histograms between the two systems mostly overlap, we can conclude that the ESM communication is not the major contributor to accuracy in the Proof of Concept scenario.

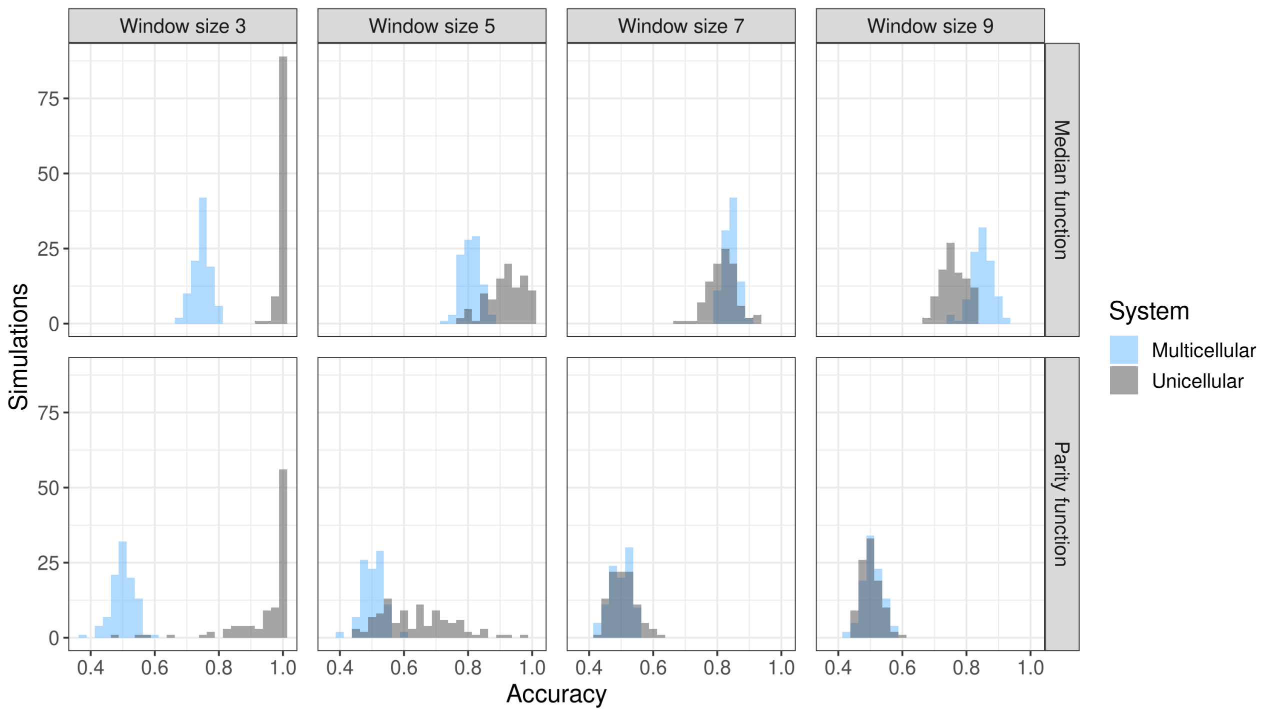

Fig. S5. Comparison between a unicellular system and a multicellular system similar to the Proof of Concept analysis. Here, we have altered the multicellular system such that one half of the strains is capable of receiving the input signal and the other half is used for output. This analysis compares a unicellular system with a multicellular system in which ESM communication is necessary to carry information about the input signal without spatial heterogeneity. Each histogram is the accuracy distribution of 100 simulations. In contrast to the other scenarios, these multicellular systems improve in accuracy as the window size increases (visible in median function), indicating that memory is an influential component. Notably, unicellular systems perform better at shorter window sizes.

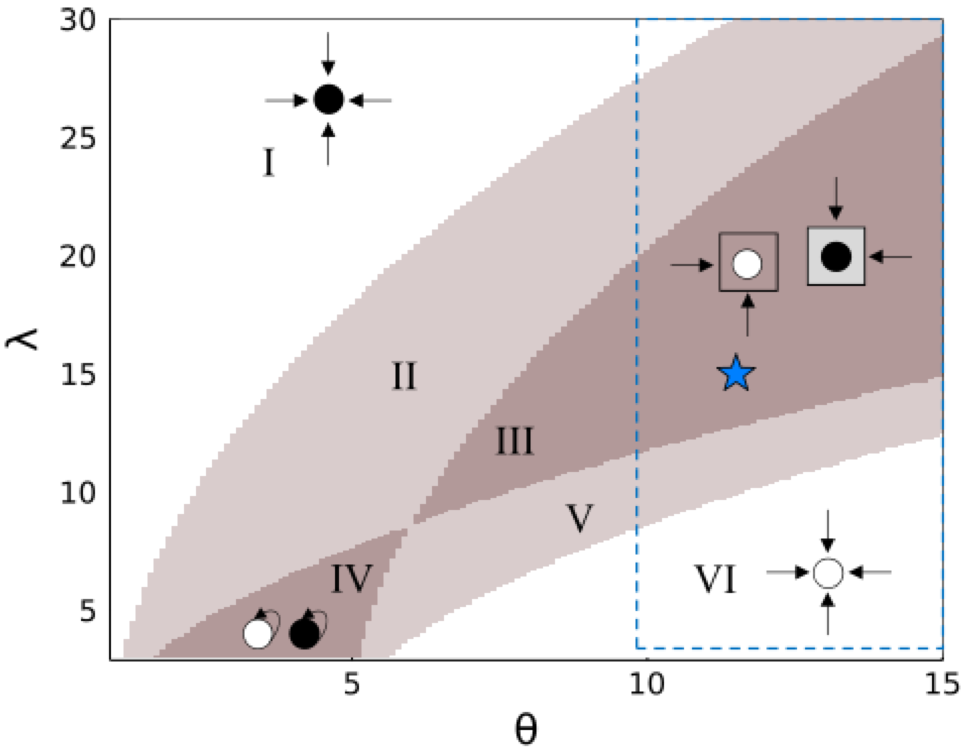

Fig. S6. Communication regions within the λ, θ parameter space. In regions I, VI, and IV no communication occurs because the ESM receptor is always on, always off, or only responds to changes in self-generated signal, respectively. In regions II, III, and V communication between cells can occur; the receptor responds to either self- or neighbor-generated signal (II), only neighbor-generated signal (III), or the necessary combination of both self- and neighbor-generated signal (V). The blue star represents the λ, θ values chosen for the Proof of Concept and Layered Community analysis. Here, cells are communicating while limiting strong signal diffusion to the neighboring cells. The blue dotted box represents the range of parameter values used for Sensitivity Analysis. Within this range, all communication regions except IV are represented. Note that the range of θ extends beyond the plot ($\theta = 10 : 100$), but are not shown here to give a better view of the communication regions. This figure was originally published in Echlin(2019)(2) and has been superficially modified here.

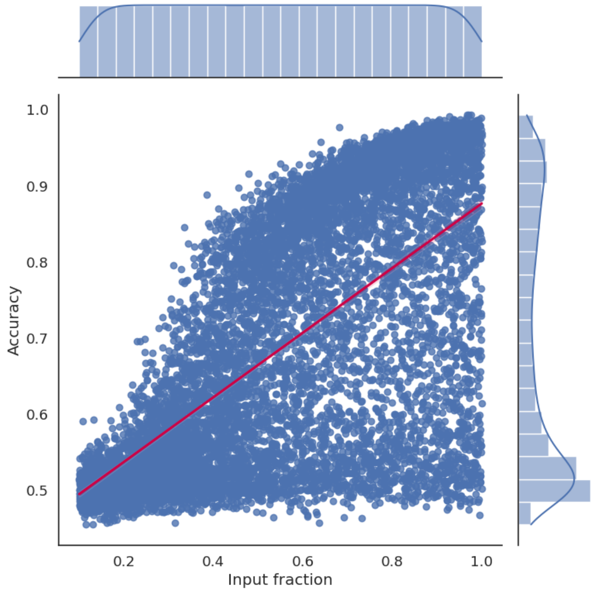

(a) Input fraction correlation

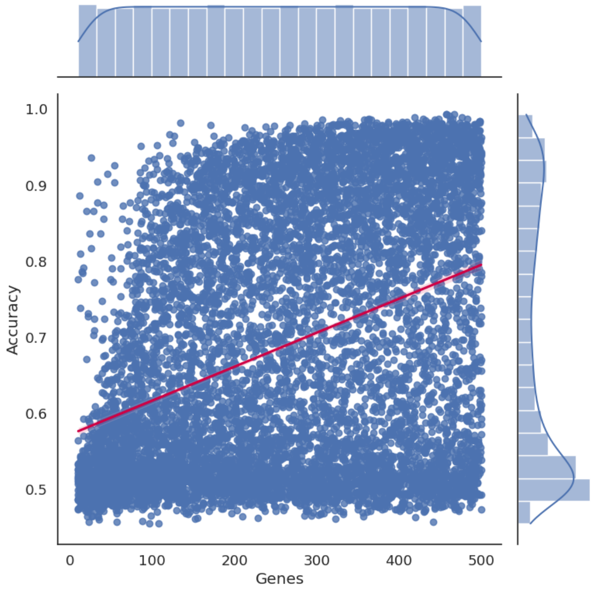

(b) Genes correlation

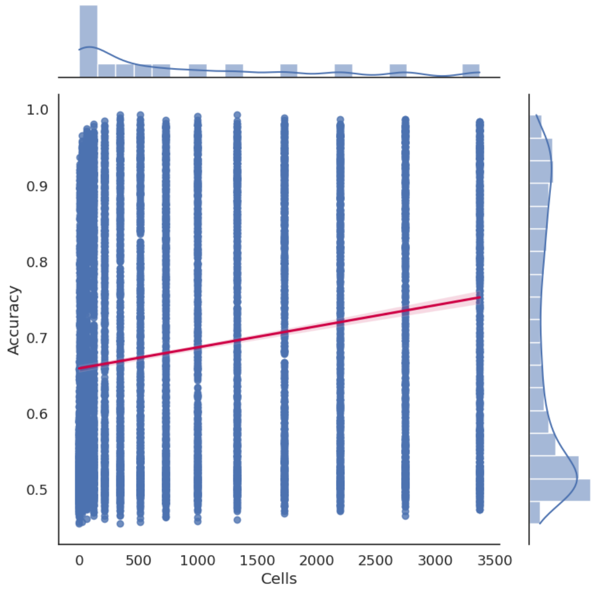

(c) Cells correlation

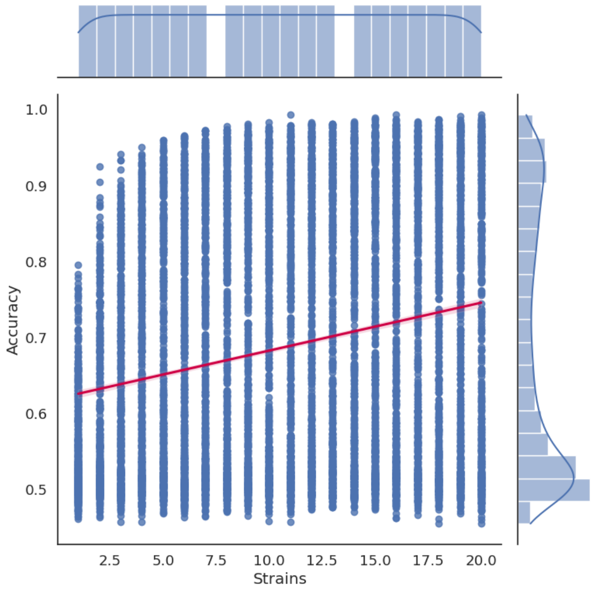

(d) Strains correlation

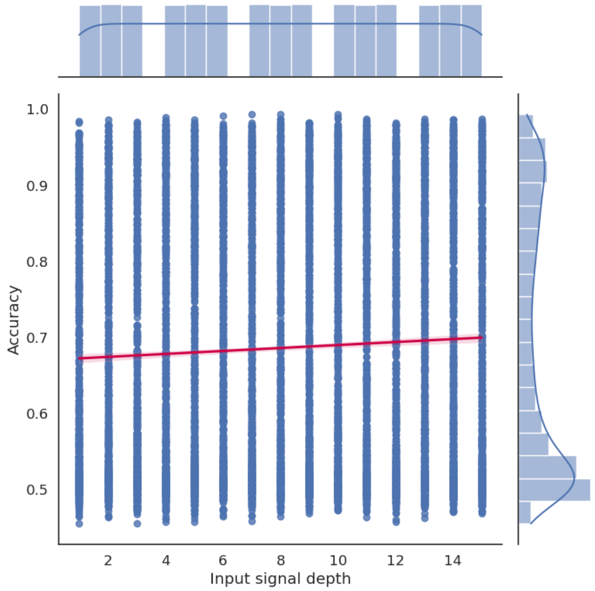

(e) Input layers correlation

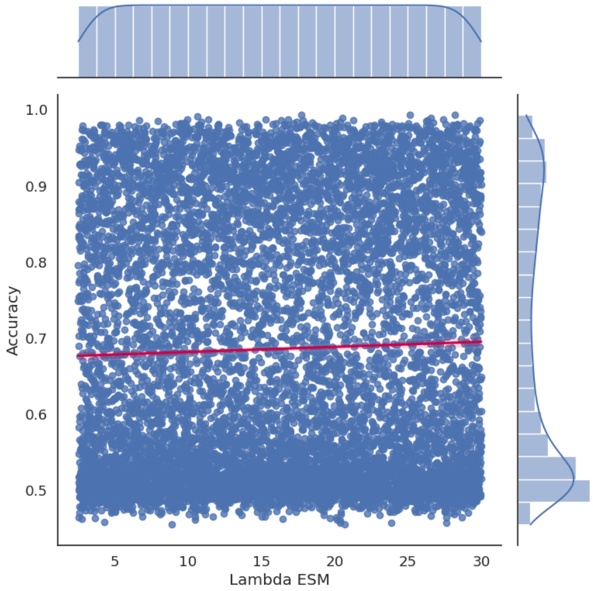

(f) ESM λ correlation

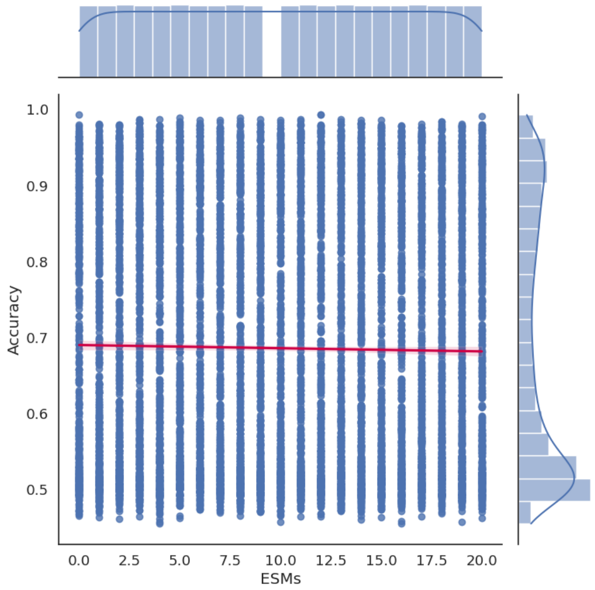

(g) ESMs correlation

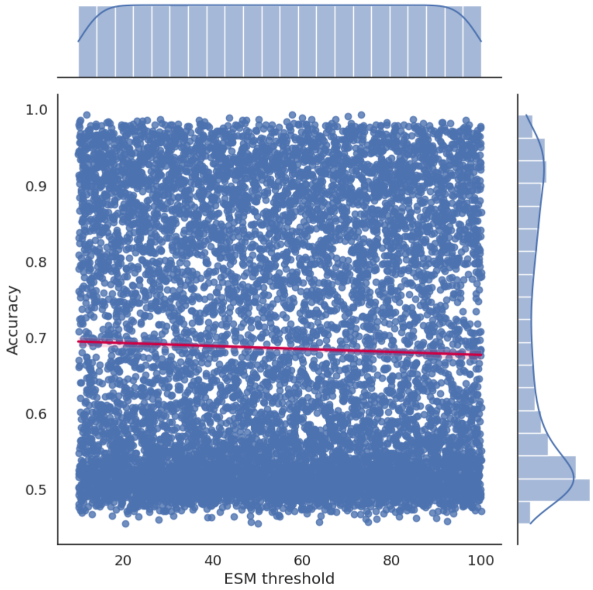

(h) ESM θ correlation

Fig. S7. Sensitivity Analysis correlation plots for every tested parameter. The red line shows a linear regression fit. A total of 50,000 simulations were done.

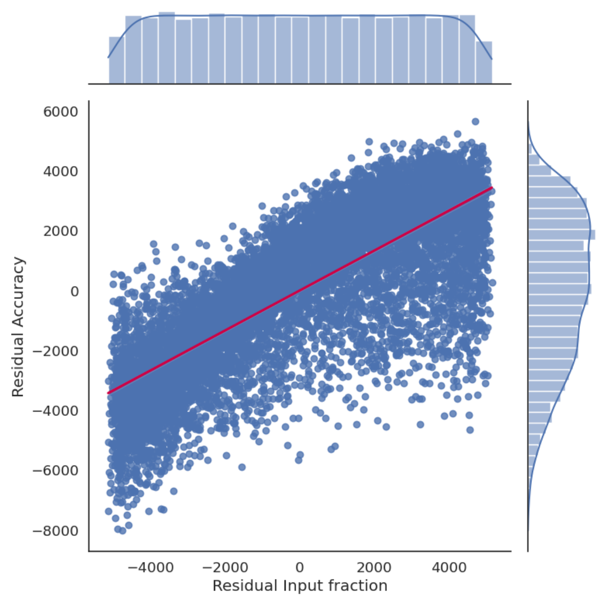

(a) Input fraction partial correlation

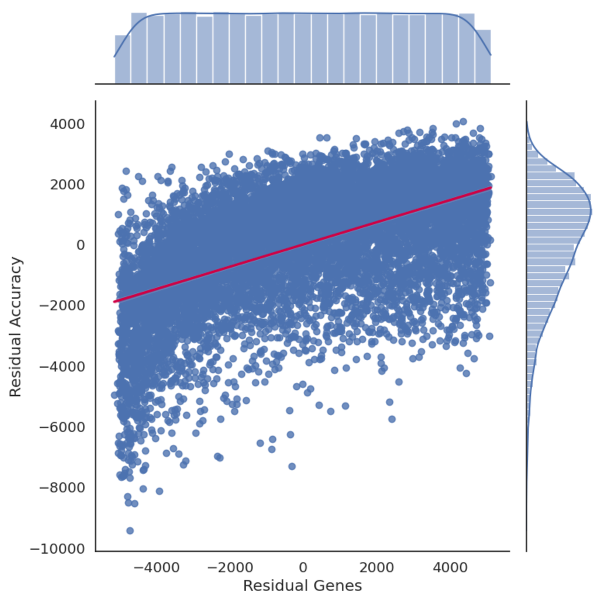

(b) Genes partial correlation

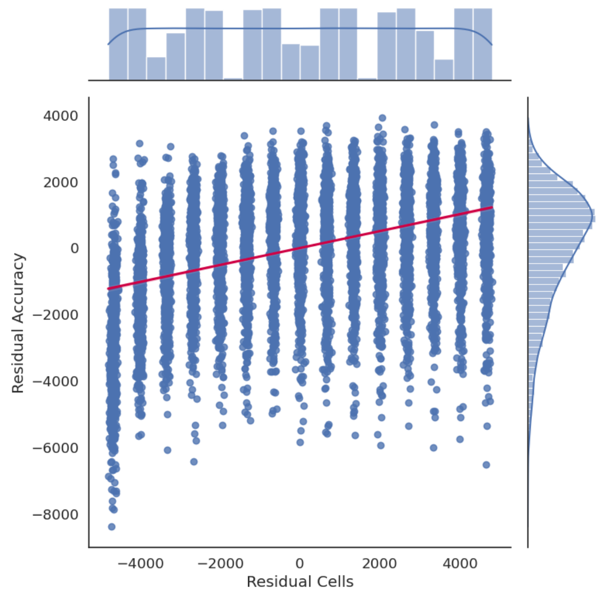

(c) Cells partial correlation

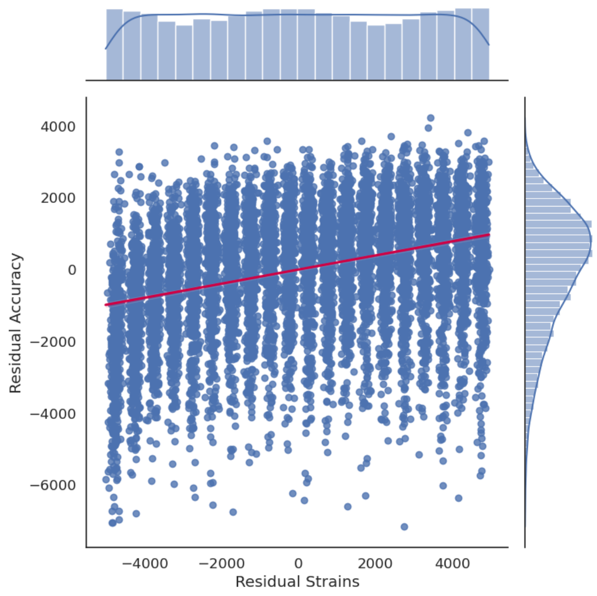

(d) Strains partial correlation

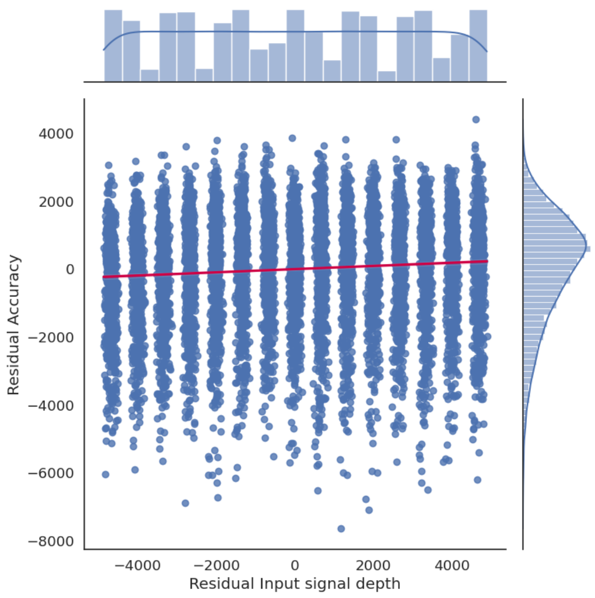

(e) Input layers partial correlation

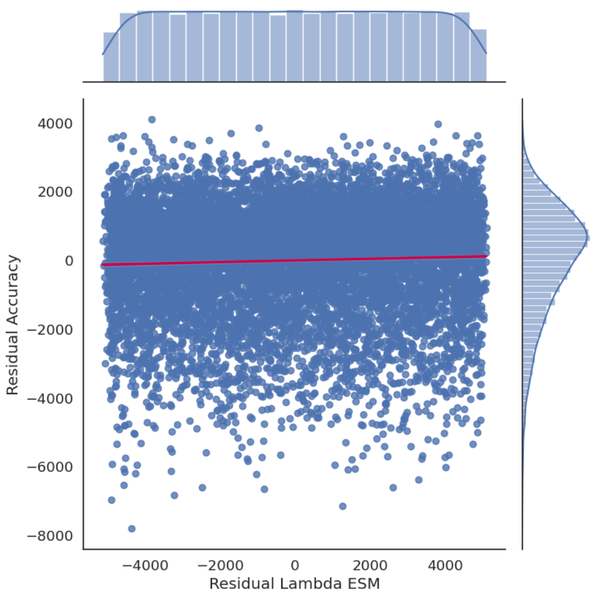

(f) ESM λ partial correlation

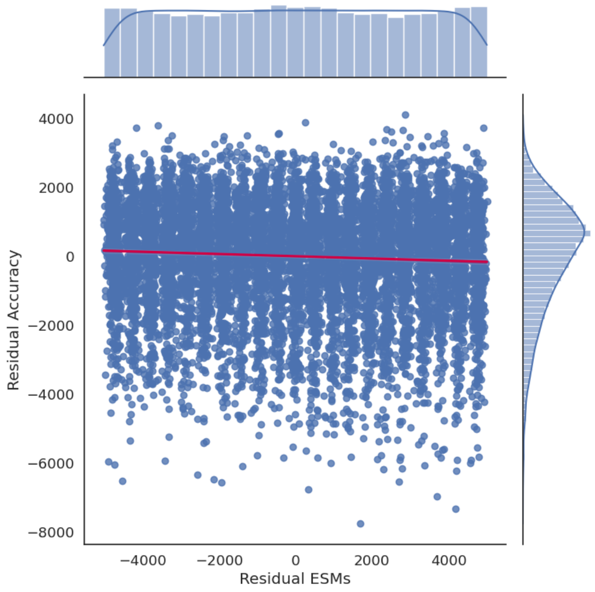

(g) ESMs partial correlation

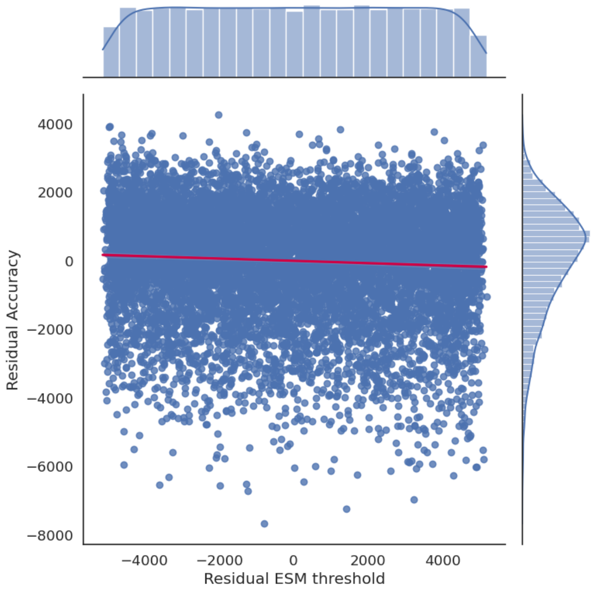

(h) ESM θ partial correlation

Fig. S8. Sensitivity Analysis partial correlation plots for every tested parameter. The red line shows a linear regression fit. A total of 50,000 simulations were done.

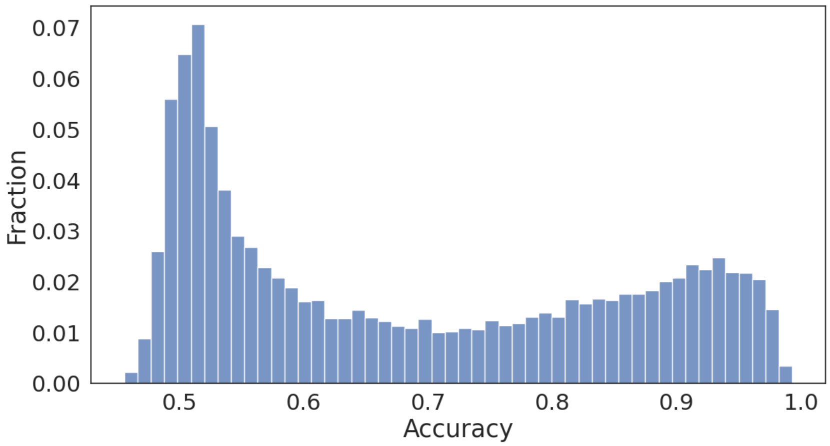

Fig. S9. Simulation accuracy results for Sensitivity Analysis. Each of the 10,000 sets of parameters was simulated 5 times for a total of 50,000 simulations. The values for fixed parameters were chosen in a manner that would evenly distribute the accuracies between the 0.5 to 1.0 range.

References

[1] Moriah Echlin, Boris Aguilar, Max Notarangelo, David L. Gibbs, and Ilya Shmulevich. Flexibility of boolean network reservoir computers in approximating arbitrary recursive and non-recursive binary filters. *Entropy*, 20(12), 2018. ISSN 1099-4300. doi: 10.3390/e20120954. URL https://www.mdpi.com/1099-4300/20/12/954.

[2] Moriah Echlin. *A Complex Systems Approach to Understanding Cells as Systems and Agents*. PhD dissertation, University of Washington, August 2019.
